# Evaluation of a person-centred, group-based, culturally appropriate diabetes education model for migrants with type 2 diabetes: a qualitative study

**DOI:** 10.1017/S1463423625100595

**Published:** 2025-12-05

**Authors:** Emina Hadziabdic, Katarina Hjelm

**Affiliations:** 1 Department of Public Health and Caring Sciences, Uppsala University, Uppsala, Sweden; 2 Department of Health and Caring Sciences, Linnaeus University, Växjö, Sweden

**Keywords:** Culturally appropriate diabetes education model, Evaluation, migrants, nursing, person-centred group-based, type 2 diabetes

## Abstract

**Background:**

Diabetes mellitus is a prevalent chronic illness worldwide and largely impacts migrants who have settled in developed countries. In diabetes care, patients play a central role and are natural partners in self-care education for improving health. Upon reviewing the literature, no studies were found that evaluated culturally adapted education models led by a nurse and delivered by a multi-professional team from the perspective of migrants in a group setting. Therefore, this study aims to explore patients’ evaluation of the content and implementation of a person-centred, group-based diabetes education model for migrants with type 2 diabetes led by a nurse and delivered by a multi-professional team.

**Method:**

Qualitative exploratory study, using semi-structured interviews in focus groups and individually to collect data. Eleven migrants who had participated in an intervention testing the education model aged 45–70, who had been living in Sweden between 4–32 years participated. Inductive qualitative content analysis of data was undertaken.

**Results:**

Participants gave a positive picture of their experiences concerning the content and organisation of the person-centred, group-based, culturally adapted diabetes education model. The education sessions were described as providing new and evidence-based knowledge. The multi-professional education staff and the interpreter were perceived as having a professional and familiar approach. They wanted to recommend the education model to others.

**Conclusions:**

The study revealed a well-functioning diabetes education model tailored to individual beliefs and cultural aspects. It improved perceived knowledge about type 2 diabetes among migrants, thus increasing self-care behaviour and health. In today’s multicultural society, the study offers insights into migrants’ feelings, ideas, concerns, knowledge, and experience regarding the content, structure, and outcome of a group-based, culturally adapted diabetes education model that can improve self-care behaviour to promote health and prevent illness. As a result, the education model can be used in primary healthcare as a central and natural partner in self-care education to improve health.

## Background

Diabetes mellitus is one of the most common chronic illnesses in the world. Type 2 diabetes forms about 85%–90% of all cases involved with diabetes and mainly affects migrants living in developed countries (International Diabetes Federation, 2021). Lifestyle changes due to migration can negatively affect health, as changing health behaviours and cultural practices often lead to unhealthier lifestyles, particularly in diet and weight gain. Culturally sensitive interventions designed to promote healthy behaviours are key to reinforcing the positive effects of the acculturation process while modifying potential negative effects, such as decreasing complications from chronic illness disease and decreasing co-morbidities (Spadea *et al.*, 2018). However, the literature review did not reveal any studies of culturally adapted education models being group-based for migrants with diabetes led by a nurse delivered by a multi-professional team and evaluated from the migrant patients’ perspective. Therefore, the present investigation aims to explore patients’ evaluation of the content and implementation of a person-centred, group-based diabetes education model for migrants with type 2 diabetes led by a nurse and delivered by a multi-professional team. The users’ perspective is important to improve the diabetes education model, enhance overall health and well-being for migrants with type 2 diabetes, and prevent complications from type 2 diabetes.

In recent years, indications have suggested that the most crucial key to good diabetes care is the patient’s active participation in self-care, based on the person’s knowledge of the disease and the body. Patient education in self-care plays a fundamental role in managing individuals with diabetes. This education can be delivered either individually or in groups. According to the Swedish National Guidelines for Diabetes Care, healthcare services should offer group-based patient education led by a nurse with both expertise and pedagogical skills to achieve optimal treatment outcomes (The National Board of Health and Welfare, 2018). In addition, lifestyle interventions, including community-based programmes and culturally tailored education, may improve glycaemic control, leading to decreased complications from Type 2 diabetes and preventing the risk of cardiovascular diseases, thereby contributing to better overall health and well-being among the migrant population with type 2 diabetes (Rawal *et al.*, 2021, Rahim *et al.*, 2024, Althubyani *et al.*, 2024, The National Board of Health and Welfare, 2018). In diabetes care, the patient has a central role and is a natural partner who needs education in self-care. Therefore, education is one of the essential aspects of care for diabetes because it improves knowledge about the disease, thus enhancing the patient’s self-care and improving health outcomes (Miller *et al.*, 2020, Chatterjee *et al.*, 2018, Brown *et al.*, 2019). A previous study (Goff *et al.*, 2020) investigated healthcare practitioners’ experiences providing diabetes self-management education and support (DSMES) to African and Caribbean adults living with type 2 diabetes and found that healthcare practitioners lacked confidence in delivering diabetes self-management education support to black African and Caribbean communities. Additionally, they experienced that existing diabetes education was generic and did not align with the specific needs of patients from ethnic minority backgrounds (Goff *et al.*, 2020). Previous studies have shown dissimilarities in beliefs about health and illness in persons of different foreign origin compared to Swedish-born people diagnosed with diabetes mellitus influencing health-related behaviour and care-seeking (Hadziabdic and Hjelm, 2022, Hadziabdic and Hjelm, 2023, Hjelm *et al.*, 2003, Hjelm and Bard, 2013, Hjelm *et al.*, 2005, Hjelm *et al.*, 1999). It has been recommended that the culturally adapted education model for migrants should be tailored to individual health and illness beliefs and knowledge to improve diabetes self-management among migrants (Hadziabdic *et al.*, 2020). A previous review highlighted barriers to DSMES for under-represented groups and minority ethnic groups with type 2 diabetes mellitus. It revealed that DSMES programmes may not consistently be tailored to the cultural and language needs of these groups and that issues like inconvenient timing and location of DSMES sessions, as well as stigma and lack of healthcare trust, can discourage participation in DSMES (Hadjiconstantinou *et al.*, 2021). However, few studies have been found adapting culturally appropriate health education for people diagnosed with type 2 diabetes in ethnic minority groups (Attridge *et al.*, 2014, Creamer *et al.*, 2016, Gamble *et al.*, 2017, Hawthorne *et al.*, 2008) and a previous study has explored the impact of a culturally sensitive DSMES programme on mental and physical health in immigrants (speaking Urdu, Arabic and Turkish) in Denmark (Hempler *et al.*, 2023). The study found that the culturally sensitive DSMES effectively improved health outcomes for the immigrants investigated. In summary, investigations on evaluations from the immigrants’ perspective are needed.

### A person-centred, group-based, culturally appropriate diabetes education model

A previously developed person-centred, group-based diabetes education model for migrants with type 2 diabetes, by the research group, is evaluated in this study (for details, see Hadziabdic *et al.*, 2020). It is a culturally appropriate diabetes education model that proceeds from individual beliefs about health and illness based on knowledge. It is conducted in focus group discussions (4-5 persons/group) in five sessions and in the presence of a professional interpreter. Further, the education model is led by a diabetes specialist nurse in collaboration with a multi-professional team and completed within three months (Hadziabdic *et al.*, 2020). The topics discussed in the educational sessions are presented in Table 1.

**Table 1.** Overview of the culturally appropriate diabetes education model led by a nurse and delivered by a multi-professional team

Session and length of session	1:st session about 90 min/session	2:nd session about 90 min/session	3:rd session about 90 min/session	4:th session about 90 min/session	5:th session about 90 min/session
Content for discussion	Perceptions of illness, the discovery of diabetes, its effects on the body, and future health.	Focuses on attaining optimal blood sugar control through diet, exercise, medication, and knowledge.	Addresses diabetes-related complications, their impact, and preventive measures, with a specific emphasis on foot care	Explore dietary habits and fundamental principles for dietary adjustments to achieve optimal blood sugar control	Summarises the methods for achieving good blood sugar control through a combination of diet, exercise, medication, and knowledge and also provides a platform for addressing participants’ inquiries
Teaching method	Focus-group interviews proceed from individual beliefs about health and illness based on knowledge and are conducted in the presence of a professional interpreter				
Group size	4-5 persons/group				
Total duration for education	About three months				

The diabetes education model implies an educational approach considering the intricate interplay of cultural and religious beliefs, gender dynamics, socio-economic backgrounds, and language disparities. This multifaceted model aims to improve diabetes management and glycaemic control to prevent diabetes-related complications by addressing the diverse needs of individuals and communities affected by this chronic condition (Hadziabdic *et al.*, 2020). It provides information to the same gender in groups and also adapts food advisories to adapt to the requirements of a particular society (Attridge *et al.*, 2014). The diabetes education model is tailored to individual and cultural aspects and can improve knowledge about type 2 diabetes among migrants, thus increasing self-care behaviour and improving health (Hadziabdic *et al.*, 2020). However, a literature review has not shown any studies within the context of person-centred culturally adapted education models being group-based, led by a nurse, and delivered by a multi-professional team, evaluated from the perspective of migrants. It is crucial to explore patients’ evaluation of the content and implementation of a person-centred, group-based diabetes education model for migrants with type 2 diabetes led by a nurse and delivered by a multi-professional team.

## Methods

This qualitative exploratory study collects data through semi-structured interviews, as the field is scarcely studied and this form of interviews allows participants to share their experiences while adhering to a predefined framework (Patton, 2015). All but two interviews were held in focus groups as planned. Due to private reasons (illness, travelling abroad), two persons could not attend the focus group they belonged to in the diabetes education and, thus, were interviewed individually. Focus group interviews are used to encourage participant engagement and gain deeper insights into their beliefs and experiences, and have been considered appropriate for verbalisation and identification of different beliefs, experiences and ideas (Krueger and Casey, 2015). Group interaction facilitates the respondents’ ability to express and clarify their beliefs. Also, it encourages participants to disclose behaviour and attitudes that might not consciously be revealed in one-on-one situations. The technique has been considered particularly appropriate in verbalising different cultural beliefs and values, emphasising the participants’ perspective (Krueger and Casey, 2015). A small group design (3–5 persons/focus group) was chosen for practical reasons, making the group manageable within a reasonable time frame and in the presence of the moderator and an interpreter, and giving all participants the same chance to let their voices to be heard (Traynor, 2015). When the aim of a study is exploratory, it has been recommended to run groups with smaller sizes, as the prime aim is to get the maximum amount of information (Traynor, 2015).

### Setting

The education model to be evaluated was conducted in primary healthcare centres (PHC) located in immigrant-dense areas in a city in Sweden. The city had approximately 245,000 inhabitants, of whom 29,6% were born abroad (Statistics of Sweden, 2023). The PHC setting is the cornerstone of the health care system and is in charge of public health and handling illnesses and injuries that do not require hospital or specialist care, e.g. management of type 2 diabetes. PHC is arranged by healthcare centres, with outpatient clinics staffed by general practitioners (GPs) and diabetes specialist nurses. Each PHC area operates a specified population based on locality and access criteria. Patients needing specialised care are referred to hospitals, with the most highly specialised care provided by university hospitals (The National Board of Health and Welfare, 2020).

The evaluation followed the last (fifth) session of the person-centred, group-based, culturally appropriate diabetes education model developed by the research group Hadziabdic *et al.*, (2020). A diabetes specialist nurse headed it. The education had been implemented in collaboration with diabetes team members (physician, dietician, and diabetes specialist nurse) at the PHC centres. The education included five planned sessions implemented in focus groups of four or five migrants diagnosed with type 2 diabetes.

### Participants

Participants in this study had been recruited to participate in an intervention study testing the diabetes education model. Thus, they agreed to evaluate the content and implementation after it was completed. Permission for the study to be executed was given by the operation managers of the PHC centres after the researcher’s contact and information (oral and written) about the study.

Upon obtaining the necessary approval, the operation managers collaborated with diabetes specialist nurses to identify individuals who met the inclusion criteria, utilising digital records at the centre. Inclusion criteria to be eligible to participate in the education were people with migrant backgrounds, aged above 18 years, diagnosed with type 2 diabetes (ICD E 11) (World Health Organisation, 2022) and with a duration of the disease of at least one year. Persons with known psychiatric diagnoses (ICD F 00-F29/F60.F 99) registered in the medical record were excluded because cognitive deficiency might influence the results. Identified participants were sent or given an invitation letter with information about the group-based diabetes education and the evaluation study and filled in a reply coupon returned to staff in the healthcare centre, then forwarded to the researcher. The invitation letter was translated by authorised translators into the language spoken by the individual. Data were collected following the completion of the education model. Hence, the evaluation involved eleven participants as a result of multiple dropouts attributed to private reasons, such as family matters, illness and unplanned travel.

The study population included eleven migrants—four females and seven males—aged 45 to 70 years (median 57 years). Their residence time in Sweden was four to 32 years (median 10 years, see Table 2), and they originated from Iraq, Lebanon, Somalia, Syria, and Sudan.

**Table 2.** Characteristics of the study population

Variable	Total n
Gender	
Male	7
Female	4
Age^1^	57 (45–70)
Country of birth	
Syria	5
Iraq	2
Somalia	2
Lebanon	1
Sudan	1
Time of residence in Sweden^1^	10 (4–32)
Reason for migration	
Refugee	9
Labour	0
Family ties	2
Duration of diabetes	17 (3–37)
Diagnosis of DM in Sweden	3
Diagnosis of DM abroad	8
Treatment	
Diet	1
Oral agents	10
Insulin	0
Combination	0
Self-reported complications	
Eye	7
Kidney	1
Heart	1
Lower extremity	0
Other	2
Educational level	
None	1
Elementary school ≤6 years	1
Primary school ≤9 years	6
Upper secondary school	2
University <2 years	0
University ≥2 years	1
Employment status	
Students	3
Gainfully employed	1
Unemployed	4
Retired	3
Marital status	
Married	10
Widow	1
Divorced	

^1^ Values are Median (range).

### Data collection

Data were collected from August 2022 to May 2023 through four focus groups, each consisting of two to three participants and two individual interviews. The interviews were conducted by two experienced diabetes specialist nurses with extensive experience in leading diabetes education, both individually and in groups. The interviews were held in a secluded location at the primary healthcare centres, lasted from 12 to 40 minutes (median 18 minutes), and had round-table seating arrangements in focus groups to facilitate interaction in the group (Krueger and Casey, 2015). Interviews were held in the presence of a professional authorised interpreter who interpreted literally, using the first person (I-form), was neutral and maintained confidentiality (Kammarkollegiet, 2019).

The interviews were performed using a thematised interview guide with open-ended questions. The interview guide was designed to evaluate a person-centred, group-based education programme starting from the individual’s beliefs about health and illness and based on experiences from evaluations of diabetes care from the patient’s perspective (Hjelm *et al.*, 2007, Hjelm *et al.*, 2009, Hjelm *et al.*, 2002). The interview guide was also discussed and peer-reviewed by a multi-professional diabetes team in primary healthcare experience in the management of individuals with type 2 diabetes.

The interview guide included the central areas of the migrants’ evaluation of the person-centred, group-based education programme, the process or content of the education, the organisation or structure of the education and the outcome regarding the person’s emotions and attitudes to the person-centred, group-based education programme. Also, follow-up questions were posed, for example: If so, what can be changed/improved? If not, what is missing? The interview guide was pilot-tested in the first focus group, and some minor changes were made concerning the wording and sequencing of the questions.

Eleven individuals participated in the evaluation and were interviewed in the four focus groups (two or three persons per group). Two individual interviews were conducted due to participants’ practical reasons, such as planned travel. The size of focus groups and the implementation of focus groups are based on gender and language considerations to promote a comfortable group dynamic (Krueger and Casey, 2015).

The focus group sessions were characterised by lively interaction, and in the context of individual interviews, communication was free-flowing and proceeded uncomplicated. All interviews were audio-recorded, and verbatim transcribed by a professional secretary (Krueger and Casey, 2015).

### Data analysis

Inductive qualitative content analysis was used to analyse data from the interviews to identify patterns in the data and discover relationships between experiences (Patton, 2015; Krueger & Casey, 2015). Following the interviews, the interviewers took reflective notes on the topics discussed, the communication with the respondents, and the group dynamics in the focus groups (Krueger and Casey, 2015). The interview transcriptions were read through as a whole and were thoroughly reviewed multiple times to gain a comprehensive understanding of the content (Krueger and Casey, 2015, Patton, 2015). The text in each content area was divided into meaning units, and each meaning unit was coded and grouped. Finally, the codes were compared for differences and similarities and sorted into sub-categories and categories. Throughout the analysis process, the authors sought to identify consistencies, contradictions, and patterns, continuously revisiting the data and transcripts until no new information was discovered. Categories were, thus, inductively created, adjusted, and refined until a satisfactory system was established, using concepts closely reflecting the text. The data analysis continued until no new information was added to the results (redundancy level) (Patton, 2015, Krueger and Casey, 2015).

As in previous research (Hjelm *et al.*, 2007, Hjelm *et al.*, 2009, Hjelm *et al.*, 2002) the evaluation and thus the results were organised around the central elements of the evaluation procedure: process (content), structure (organisation), and outcome in terms of persons’ attitudes/feelings towards the person-centred, group-based, culturally adapted diabetes education model. Data are presented as categories with sub-categories, illustrated by illuminative quotations (Krueger and Casey, 2015, Patton, 2015).

### Trustworthiness

To enhance the trustworthiness of this study, the following steps have been taken(Patton, 2015): (1) The first author analysed the data and created the codes and categories that were reviewed and assessed for relevance by the co-author (KH) to ensure credibility. If needed, the researchers discussed the results to address potential disagreements until a consensus was reached. To further increase credibility, data collection and analysis proceeded until no new information was added (redundancy level attained). This was reached after three focus-group interviews, as Krueger and Casey (2015) advocate often happens: (2) Confirmability was ensured by presenting categories, with sub-categories, illustrated by illuminative quotations and by naming categories as closely as possible to the original text and (3) Detailed descriptions of the research process confirmed dependability, and (4) Rich descriptions of the participants’ characteristics and the setting are provided to help the reader judge the transferability of the results to other participants/contexts (Patton, 2015).

### Ethics approval and consent to participate

The study was approved by a regional Ethics Committee (Approval ID: 2014/198-31, 2018/324-32) and implemented in accordance with the Helsinki Declaration (World Medical Association, 2024), with written informed consent from the participants

## Findings

The analysis resulted in five categories with respective sub-categories (see Table 3). These were organised around the central element of the evaluation procedure: process (content), structure (organisation), and outcome in terms of persons’ attitudes/feelings towards the person-centred, group-based, culturally adapted diabetes education model. Most participants gave a positive picture of their experiences of the content and organisation of the person-centred, group-based, culturally adapted diabetes education model. The education sessions were described as providing new and evidence-based knowledge, and participants cited positive aspects such as the organisation of the education model. The education staff and interpreters communicated well and showed a professional and familiar approach. The sessions allowed sharing experiences in groups with others in the same situation.

**Table 3.** Overview of categories with sub-categories analysed from interviews by migrants

Subcategory	Category	The central elements of the evaluation procedure
Gaining new knowledge	New and evidence-based knowledge about self-care, health, health risks and seeking care about the disease	Patient’s perspective on the education model
Gaining reliable knowledge		
Meeting and getting to know healthcare professionals		
Professional competence of an interpreter	A professional approach from knowledgeable education staff	
Deep and professional knowledge of staff		
Suggestions to make changes	Suggestions for changes in education module organisation or structure	Patient’s perspective on the organisation of the education model
Satisfied with the education	Getting the intended education model	
Receiving respectful treatment from teaching staff	A humble and respectful attitude	Patients’ feelings and attitudes to the education model
To have wishes fulfilled		
No proposed changes		

### Patient’s perspective on the content of the education model

#### New and evidence-based knowledge about self-care, health, health risks and seeking care about the disease

Most participants did not encounter any issues with the content of the person-centred, group-based, culturally adapted diabetes education model. They said that they gained new and evidence-based knowledge about self-care, health, health risks and care-seeking behaviour concerning the disease adapted to their background factors. This new knowledge meant they were aware of and could manage the disease better, leading to less care-seeking behaviour. Furthermore, the education provided the opportunity to meet people with similar backgrounds while building good relationships with healthcare professionals.

*‘What was good, really, is that one gets, well,…. how to take care of your disease, that you should exercise, that you should do things that can lead to improving this disease, improving the experience with this disease.’ (I: 0095).*

*‘We received very good instruction. At the same time, how to care for yourself and make your blood counts stable. And that you should learn how to exercise…and so on.’ (I:0092)*

#### A professional approach from knowledgeable education staff

The participants experienced unproblematic contact and communication with the education staff and the professional interpreter. The staff involved in the teaching model were perceived as experts in their fields. At the same time, they complemented each other and had a respectful attitude. Furthermore, the interpreters were perceived as linguistically competent with a respectful attitude, facilitating communication.

*‘They (healthcare staff) had expert skills…And we were glad. I was delighted with their treatment and everything.’ (I:0092)*

*‘He (the interpreter) was very good really. He interpreted. He interpreted in a very good way, and he was also clear. And we thought it worked very well.’ (I0095)*

*‘Interviewer: Having the same interpreter might have been an advantage. Alternatively, maybe it is an advantage to have different ones.*

*Respondent: It doesn’t matter; you just need to understand the interpreter’s dialect or language.’ (I:0235)’*

### Patients perspective on the organisation of the education model

#### Getting the intended education model

Most participants perceived the person-centred, group-based, culturally adapted diabetes education model as well-functioning. Most were content with the expectations and the current structure and organisation of the model, citing positive aspects such as group-based learning, group composition, content relevance, efficient time management, and the availability of an interpreter for effective communication. Several respondents also preferred afternoon classes lasting 1-1.2 hours and consisting of three to four individuals.

*‘No, nothing. Everything was fine. Everything was excellent. It was good. We have received a lot of good information.’ (I: 00235).*

*‘There should be at least two to three people so that you can discuss, and bring the discussion alive. Yes… If there are five, you have to sit and listen to him and him…. It might be cumbersome… So maybe three would have been the optimal.’ (I:0095).*

*‘Interviewer: Has it been an advantage to have a topic for each occasion? You know, we’ve been concentrating… Sometimes, we just talked about the diet, and sometimes, we just talked about the blood sugar and so on. Is that a good way to do it?*

*Respondent 1: Yes, it was great.’ (I: 0235).*

#### Suggestions for changes in education module organisation or structure

However, some participants suggested changes with the to-do blood tests before and after each education session to investigate the impact of the knowledge they had received during the session on their bodies. Furthermore, some gave suggestions regarding more content on the latest research in medical advances in the disease field and expressed the desire for a larger group (not three or four) to be able to learn from others in a similar situation.

*‘… I wondered if I would have done it on my body, for example, when we take the samples with something new. Try…. And get practical answers… Practical results I mean’ (I: Group 1)*

*‘… Yes. The question was that we talked about it going, for example… Or was there any research that says you can transplant the pancreas to completely cure this disease?*

*Interviewer: Mm, you would have liked more information about that?*

*Respondent: Yes, about how far along they have really come with this research.’ (I: Group KH)*

### Patients’ feelings and attitudes to the education model

#### A humble and respectful attitude

The participants experienced the education staff’s humble and respectful attitude and felt familiar with the trainee. They felt satisfied with the staff’s competence and treatment. They wanted more people in a similar situation to attend the education module and to recommend to others among their acquaintances that they actively seek out the education module.

*‘I think we were like a family.*

*Interviewer: Does it feel good that it’s a family then?*

*Respondent: Of course! If we didn’t feel so comfortable in this teaching, in these five sessions, we wouldn’t have come. We would apologise and not come.’ (I: Group 1)*

*‘Interviewer: Would you recommend to others*

*Respondent 1: Absolutely*

*Respondent 2?: Of course. Because the knowledge is not available to everyone who is diabetic, and you need it. I have already recommended that my diabetic acquaintances attend a class. It’s very good information, and they will be well-informed… I have an acquaintance who doesn’t belong to this health centre. Is it okay if I participate?’ (I: Group 1)*

## Discussion

This study is unique in that it evaluates the content, organisation, and outcome of a person-centred, group-based, culturally adapted diabetes education model for migrants from the patient’s perspective.

### Evaluation of the content of the education model

The main results showed that the participants perceived the person-centred, group-based, culturally adapted diabetes education model as well-functioning. Group-based diabetes education is highly complex and challenging for healthcare professionals to facilitate due to variations in intended purpose, content, and format (Quiñones *et al.*, 2014). In particular, the healthcare staff involved in the teaching model were perceived as experts in their fields, as having a respectful attitude and facilitating communication. Healthcare professional skills related to problem-solving, goal-setting, and facilitating active participation and group dynamics have been identified as crucial factors supporting health behaviour changes among group participants (Nossum *et al.*, 2013). The person-centred approach in patient education implies a change from healthcare staff experts from unidirectional content transmission to listening to the patient’s beliefs and concerns. It emphasises actively incorporating participants’ experiences, concerns, needs, collaboration and empowerment (Hashim, 2017). Previously, research generally focused on outcomes of group-based diabetes programmes and less on form and content (Hempler *et al.*, 2023). When investigating the content of this education model, we found that the person-centred, group-based, culturally adapted diabetes education model increased the participants perceived diabetes knowledge, self-management skills, and self-efficacy/empowerment. This is similar to a previous systematic review of group-based diabetes self-management education programmes (DSME) for people with type 2 diabetes (Steinsbekk *et al.*, 2012) and the indigenous Pacific Peoples population residing in New Zealand (Gamble *et al.*, 2017). The healthcare staff need to have a person-centred approach in patient group education to support increased knowledge about the disease, self-management skills and care-seeking behaviour.

### Evaluation of the organisation of the education model

The study found that participants were satisfied with the current structure of the model, citing positive aspects such as group-based learning, the multi-professional diabetes team’s group composition, content relevance, efficient time management, and the availability of an interpreter for effective communication. A previous study (Hollis, Glaister, & Anne Lapsley, 2013) has found that nurses who frequently engage in diabetes self-management education may show knowledge gaps, especially in medication management. This study emphasises the importance of multi-professional diabetes teams in delivering education. Multi-professional diabetes teams involved in delivering education have been found to significantly improve glycaemic control and knowledge about diabetes and are therefore recommended (Attridge *et al.*, 2014; Creamer *et al.*, 2016).

In this study, the participants’ expectations and the current structure and organisation of the education model were in harmony. The organisation of the studied education model appears to have successfully integrated both structure and management aspects in the clinical practice. Interaction in group-based learning has previously been described as effective for ethnic minority groups with type 2 diabetes (Attridge *et al.*, 2014, Brunk *et al.*, 2015, Chatterjee *et al.*, 2018, Creamer *et al.*, 2016, Gamble *et al.*, 2017, Hawthorne *et al.*, 2008) and as offering a possibility to modify beliefs about health and illnesses and illness experiences for migrants in the management of diabetic foot (Hjelm *et al.*, 2002), gestational diabetes (Hjelm *et al.*, 2007) and foot ulcers (Hjelm *et al.*, 2009). Furthermore, this study showed that both well-experienced staff and the use of an interpreter for communication were the key concepts of the education model. Otherwise, communication barriers lead to disturbance during healthcare appointments, which may decrease the level of self-care and increase the level of perceived illness (Ritholz *et al.*, 2014). The current conditions of the healthcare system are intended to provide high-quality and person-centred care. Therefore, it is important to maintain quality standards and personalise care for each person to tailor group diabetes education to participants’ cultural and religious beliefs, gender dynamics, socio-economic backgrounds, and language disparities (Hadziabdic *et al.*, 2020).

### Patients’ emotions and attitudes to the education model

The participant’s attitudes towards the education model were positive. The study found that participants experienced the education staff as humble and respectful and felt familiar with them. This finding can be explained by the fact that the diabetes education model, developed by the research group (Hadziabdic *et al.*, 2020), succeeded in exploring the patient’s feelings, ideas, concerns, knowledge and experience regarding self-care, health, health risks, and care-seeking behaviour, as well as what the migrants expected from the multi-professional team.

### Strengths and limitations of the study

The strength of this study is that it is the first study to evaluate the content, structure, and outcome of a person-centred, group-based, culturally adapted diabetes education model from the migrant’s own perspective and consider its contribution to improved managing capacity in primary healthcare. Migrants are often excluded from sharing their experiences and perspectives on healthcare due to communication barriers (Whitaker *et al.*, 2022). In this study, participants had the opportunity to express their perspectives. It provided valuable insights into migrants’ feelings, thoughts, concerns, knowledge, and experiences related to a culturally adapted, person-centred, group-based diabetes education model. This approach aims to enhance self-care behaviours, promote health, and prevent illness, thereby making a significant contribution to the improvement of healthcare services and policies.

A limitation of this study could be the chosen small group design (three to five persons planned but two to three persons in reality) during the focus group interviews (Traynor, 2015). This is explained by a substantial attrition and retention rate in the intervention study due to different factors such as the COVID-19 pandemic, health status, time constraints connected with family and job responsibilities, etc. Factors related to the infrastructural, institutional, interpersonal and individual context were out of the researchers’ control. However, a group with fewer participants will have less total experience than a larger one, giving the participants enough time or chance to communicate than in larger groups. The results rest more on the interaction and involvement of the participants in each group than the actual number of participants (Krueger and Casey, 2015). In this study, there were fluent discussions and lively interactions. Further, when having an exploratory aim, groups of smaller size should be organised to reach the prime objective of obtaining the maximum amount of information (Traynor, 2015). Consequently, the effect of the small group design was minimised. Additionally, the sample comprised mostly of people who had migrated as refugees and originating from three countries in the Middle East and two countries in Africa, making them representative of the migrant population of the mid-2000s in Sweden (Statistics of Sweden, 2023).

The mix of focus group and individual interviews for data collection might have affected the findings. Using individual interviews enabled a deeper understanding of the individual’s perspectives and experiences. On the other hand, focus group interviews stimulated participants to react to what was said by others, thereby leading to deeper expressions of experiences, particularly by disclosure of experiences and beliefs not consciously revealed (Krueger and Casey, 2015, Patton, 2015). However, it has been noted that individual interviews can yield the same information as focus group interviews, although focus groups are more complicated to arrange than individual interviews and considerably more time is needed with individual interviews (Traynor, 2015). However, the influence is negligible as all but four interviews were held in focus groups.

Another limitation could be that cross-language research can potentially alter meaning (Squires *et al.*, 2020).To ensure precise transcriptions, the interviews were translated by an experienced professional interpreter. In addition, letting participants in focus groups communicate in their mother tongue with an experienced professional interpreter increased their comfort level and data quality (Squires *et al.*, 2020).

Despite the limitations, it is worth noting that the study’s main strength is that it has recorded the migrants’ own perspectives and thoughts about the organisation and content of the person-centred, group-based, culturally adapted diabetes education model. Since the results were carefully collected and analysed, the study provides a deeper understanding of the studied topic, which makes it possible to transfer the study findings to other settings or groups with similar characteristics (Patton, 2015).

## Conclusions

In conclusion, this study revealed a well-functioning diabetes education model from the migrants’ perspective. The model was tailored to individual and cultural beliefs, and it was perceived to improve knowledge about type 2 diabetes among migrants, thus increasing self-care behaviour and health. The person-centred, group-based, culturally adapted diabetes education model thus can be used in primary healthcare and extended to include persons belonging to other migrant groups to respond to the growing and diverse migrant populations affected by diabetes worldwide.

In today’s multicultural society, where type 2 diabetes is on the increase and mainly affects migrants living in developed countries, there is an increasing requirement for a well-functioning person-cantered, group-based, culturally adapted diabetes education model within primary healthcare. This study revealed that the person-cantered, group-based, culturally adapted diabetes education model represents an efficient and personal educational approach, providing insights into the emotions, ideas, concerns, knowledge, and experiences of migrants related to the content, structure, and outcomes of the educational model. Furthermore, this education model has the potential to improve self-care behaviours, promote health and prevent illness. As a result, the education model needs to be implemented in primary health care. By adopting this education model, the healthcare staff will be better equipped to meet the evolving needs of those they serve, enhancing the quality of diabetes care and the overall health outcomes.

## Data Availability

In order to protect the integrity, anonymity and confidentiality of the informants, data will not be shared.
